# Effect of alpha-tocopherol on bone formation during distraction osteogenesis: a rabbit model

**DOI:** 10.1007/s10195-011-0145-z

**Published:** 2011-07-15

**Authors:** Mustafa Kurklu, Cemil Yildiz, Ozkan Kose, Yuksel Yurttas, Ozgur Karacalioglu, Muhittin Serdar, Salih Deveci

**Affiliations:** 1 Department of Orthopaedics and Traumatology, Gulhane Military Medical Academy, Ankara, Turkey; 2 Antalya Education and Research Hospital, Orthopaedics and Traumatology Clinic, Pinarbaşi mh. 758.sk. Nazlibahce Evleri A Blok D8, Konyaalti, Antalya, Turkey; 3 Department of Nuclear Medicine, Gulhane Military Medical Academy, Ankara, Turkey; 4 Deparment of Clinical Biochemistry, Gulhane Military Medical Academy, Ankara, Turkey; 5 Department of Pathology, Gulhane Military Medical Academy, Ankara, Turkey

**Keywords:** Distraction osteogenesis, Alpha-tocopherol, Vitamin E, Free radicals, Antioxidant therapy, Fracture healing—drug effects

## Abstract

**Purpose:**

The purpose of this study was to evaluate the effects of alpha-tocopherol on distraction osteogenesis.

**Materials and methods:**

Right tibias of 30 New Zealand white rabbits were distracted at a rate of 0.5 mm/day for 20 days with a circular external fixator. Experimental group rabbits (*n* = 15) were administered i.m. 20 mg/kg/day alpha-tocopherol for 30 days. Radiographic examinations were performed at the 20th, 30th and 40th days. Bone scintigraphy was performed at the 5th and 20th days. Serum total antioxidant capacity (TAC) was measured at the 5th and 30th days. All animals were sacrificed and the right tibias of all animals were harvested for histopathologic examination at the 40th day.

**Results:**

Radiologic scores were statistically similar at the 20th day. However, the experimental group demonstrated higher radiologic scores at the 30th and 40th days. A scintigraphic baseline study at the 5th day of the study showed statistically similar osteoblastic activities in both groups. However, at the 20th day, osteoblastic activity was significantly higher in the experimental group. Serum TAC values were also significantly higher in the experimental group at the 30th day. At necropsy, histopathologic examination revealed statistically significantly higher scores in the experimental group.

**Conclusion:**

The results of this study show that alpha-tocopherol has beneficial effects on new bone formation during distraction osteogenesis.

## Introduction

The technique of “distraction osteogenesis” is frequently used in the treatment of bony loss, pseudoarthrosis, chronic osteomyelitis, limb length discrepancy, biologic reconstruction after wide tumoral resection, and deformity [1–6]. One major problem with this method, however, is the prolonged time required for the newly formed bone in the distraction gap to consolidate and become strong enough for weight-bearing [7]. Various clinical and experimental investigations have been focused on the acceleration of bone formation and consolidation, and have thereby aimed to shorten the framing time [8–11].

Distraction osteogenesis is recognized as being “intramembranous ossification,” which can be assumed to be a special form of fracture healing [12]. Fracture healing after injury involves inflammation, repair and remodeling [13]. At the inflammatory stage, polymorphonuclear leukocytes (PMNLs), macrophages and mast cells migrate into the fracture site, and osteoclasts begin to remove necrotic bone [14, 15]. Activation of PMNLs produces oxygen free radicals, which cause lipid peroxidation and are known to impair fracture healing [16, 17]. Antioxidant administration has been shown to be beneficial in suppressing the damaging effects of oxygen free radicals in cells during fracture healing [18–21].

Consequently, we hypothesized that alpha-tocopherol, which is a potent antioxidant, may also have favorable effects on the quality of new bone formation during distraction osteogenesis and shorten the time required for consolidation. Thus, in this study, the effect of alpha-tocopherol on bone formation during distraction osteogenesis was investigated.

## Materials and methods

### Animals

In this study, 30 adult New Zealand white rabbits (mean weight 1,800 g; range 1,500–2,000 g) were used. The animals were fed a standard laboratory diet and water and had a 12 h day/night cycle. The rabbits were housed separately in standard cages in a temperature-controlled room (20–22°C). Before initiating the study, approval from the local ethics committee was obtained. The study was carried out in the Center for Experimental Animals at the same institution. The rabbits were randomized into experimental and control groups each consisting of 15 animals.

### Surgical procedures (day 1)

**Fig. 1:**
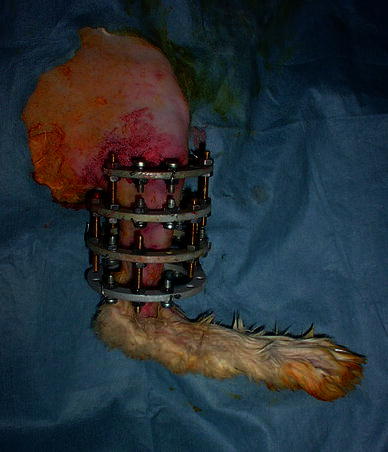
Application of the circular external fixator to the right tibia of a rabbit

Rabbits were anesthetized with 0.2 mg/kg xylazine and 20 mg/kg ketamine hydrochloride. Infection prophylaxis was provided with 20 mg/kg/day cefazoline sodium preoperatively and 2 days postoperatively. When the appropriate depth of anaesthesia had been achieved, a preconstructed four-ring circular external fixator was applied to the right tibia of each rabbit. 5/8 rings were used at the distal and proximal levels. Each ring was connected with three rods. Two Kirchner wires (1 mm in diameter) crossing at angles of 45–60° with respect to each other were applied to every level. The skin subdermis and periosteum were exposed with an anteromedial longitudinal incision, and a transverse osteotomy was performed on the middle third of the right tibia. Subsequently, the periosteum and skin were closed properly (Fig. 1). After then waiting for 5 days, distraction was applied to each tibia on a schedule of 0.125 mm per 6 h a day (0.5 mm/day). Distraction continued for 20 days.

### Experimental intervention (day 1–30)

The experimental group of rabbits received 20 mg/kg alpha-tocopherol intramuscularly starting on the first day of study, and a daily injection of alpha-tocopherol was given for 30 days thereafter. The control group did not receive any corresponding treatment.

### Radiological follow-up and evaluation (days 20, 30 and 40)

Radiographs were taken at the 20th, 30th and 40th days of the study and evaluated by one of the investigators (who was blinded to the assignment of study groups) using the five-point grading scale described by Lane and Sandhu (Table 1) [22].

**Table 1:** Radiologic evaluation system [22]

Bone formation	Score
Lack of bone formation	0
Bone formation filling 25% of the defect	1
Bone formation filling 50% of the defect	2
Bone formation filling 75% of the defect	3
Bone formation filling 100% of the defect	4
Union distal	
No union	0
Onset of union	1
Complete radiologic union	2
Union proximal	
No union	0
Onset of union	1
Complete radiologic union	2
Remodeling	
None	0
Formation of intramedullary channel	1
Formation of cortex	2
Total score (maximum)	10

### Scintigraphic method (days 5 and 20)

Before the scintigraphic assesment, the rabbits were sedated with 10 mg/kg ketamine hydrochloride. In the scintigraphic study, 3 ± 0.5 mCi/0.5 cc technetium–99 m methylene diphosphonate was injected into the ear vein of each rabbit. Three hours after the injection of the radiopharmaceutical, the subject was positioned laterally under the gamma camera (Millenium, General Electric, Milwaukee, WI, USA) equipped with a low energy, high-resolution collimator, and planar acquisition was initiated for 10 min using a 15% window centered over the 140 keV photopeak. Rectangular regions of interest (ROIs) were drawn on both tibias (the region of distraction osteogenesis and the contralateral healthy leg) at approximately similar locations. Counts were derived from both ROIs in order to calculate the osteoblastic activity ratio (count for the lesion/count for the contralateral side). Scintigraphic assessments were performed on the 5th and 20th days of the study, before and after the distraction period.

### Total antioxidant capacity (TAC) measurement (days 5 and 30)

Plasma TAC was measured using a Randox total antioxidant status kit (Total Antioxidant Status, Randox, Crumlin, UK) in which ABTS (2,2-azino-di-[3-ethylbenzthiazolin-6-sulfanate]) is incubated with a peroxidase and H_2_O_2_ to produce the radical cation ABTS+. This has a stable blue-green color, which is measured at 600 nm. Antioxidants present in the sample suppress this color production to an extent that is proportional to their concentration. The suppression of the absorbance of the ABTS+ radical cation by serum antioxidants was compared with the suppression caused by Trolox (6-hydroxy-2,5,7-tetramethylchroman-2-carboxylic acid), which is included as part of the TAC kit. The results are expressed as mmol/l of the Trolox equivalent [23].

### Histopathologic evaluation (day 40)

All rabbits in each group were sacrificed with a high-dose intraperitoneal sodium pentobarbital injection on the 40th day of the experiment. After sacrifice, all tibias were harvested, stripped from their soft tissue, and sent to a pathologist, who was unaware of the allocation of rabbits into groups. For histological evaluation, extremities dissected from their soft tissues were fixed in 10% neutral buffered formaldehyde solution for ten days, and then treated with a rapid decalcification solution (formic acid) for five days. After the decalcification procedure, tibias were sampled longitudinally from distracted segments. For each tibial segment, four cuts were taken from the distraction area and routine histological preparation was carried out. Samples were embedded in paraffin, and sections of 4 μm width were taken from the paraffin blocks and stained with hematoxylin–eosin. Sections were examined under a light microscope. Histopathological evaluation was done according to the grading system described by Huddlestone et al. (Table 2) [24].

**Table 2:** Histopathologic evaluation system [24]

Tissue	Score
Fibrous tissue	1
Predominantly fibrous tissue with some cartilage	2
Equal amounts of fibrous and cartilage tissue	3
All cartilage	4
Predominantly cartilage tissue with some woven bone	5
Equal amounts of cartilage and woven bone	6
Predominantly woven bone with some cartilage	7
Entirely woven bone	8
Woven bone and some mature bone	9
Lamellar (mature) bone	10

### Statistical analysis

The Wilcoxon signed-rank test was used for repeated measurements of the same group and the Mann–Whitney *U* test was employed to compare groups used. Statistical significance (*P* < 0.05) was determined based on the 95% confidence interval.

## Results

All rabbits in both groups completed the experiment without any complications, and the pertinent results are summarized in Table 3.

**Table 3:** Summary of results (mean radiologic and histopathologic scores, TAC values, and scintigraphic counts for the groups, as well as statistical significance)

	Experimental group (SD)	Control group (SD)	*P* value
5th day TAC value (mmol/L of Trolox equivalents)	2.58 ± 0.28	2.59 ± 0.14	0.389
30th day TAC value (mmol/L of Trolox equivalents)	2.75 ± 0.28	2.50 ± 0.08	0.001
5th day scintigraphic ROI (distracted/normal, ratio)	1.06 ± 0.20	0.97 ± 0.18	0.233
20th day scintigraphic ROI (distracted/normal, ratio)	3.63 ± 1.06	1.65 ± 0.54	0.000
20th day X-ray score	1.73 ± 0.45	1.46 ± 0.51	0.217
30th day X-ray score	4.60 ± 0.91	3.46 ± 0.83	0.003
40th day X-ray score	7.73 ± 1.09	5.46 ± 0.83	0.000
40th day histopathological grade	9.86 ± 0.35	8.00 ± 0.92	0.000

*SD* standard deviation

### Radiologic results

**Fig. 2:**
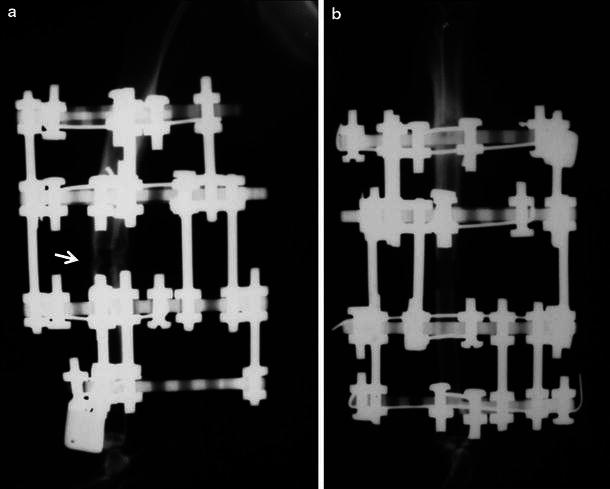
X-ray of a rabbit in the experimental group (**a**) and in the control group (**b**) on the 40th day of the study. Note the enhanced callus formation and consolidation in the experimental group (*arrow*)

On the 20th day, radiologic scores were statistically similar in both groups (*P* = 0.217). However, on the 30th and 40th days, the experimental group displayed statistically significantly higher radiologic scores and visible callus formation, maturation and remodeling (*P* = 0.003 and *P* = 0.000 respectively; Fig. 2).

### Scintigraphic results

Scintigraphic baseline study on the 5th day of study showed statistically similar osteoblastic activities in both groups (*P* = 0.233). However, on the 20th day, the osteoblastic activity was significantly higher in the experimental group (0.000).

### TAC measurement results

Serum TAC values were statistically similar in both groups on the 5th day of the study (*P* = 0.389). However, on the 30th day, TAC values were significantly higher in the experimental group (*P* = 0.001).

### Histopathologic results

**Fig. 3:**
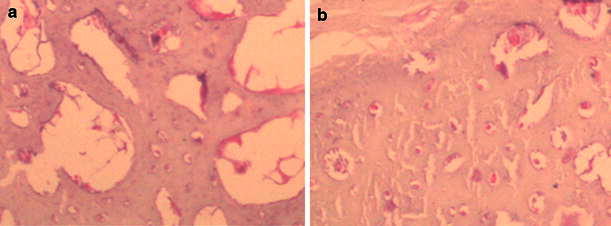
Histopathologic examination of a rabbit in the experimental group (**a**) and in the control group (**b**). Note the mature lamellar bone in the experimental group and the predominantly woven bone with some cartilage in the control group (H&E stain, original magnification ×50, light microscopy)

At necropsy, rabbits in the experimental group had statistically significantly higher scores in the histopathologic examination, and showed the formation of mature bone (*P* = 0.000; Fig. 3).

## Discussion

In this study, possible favorable effects of alpha-tocopherol on the quality of new bone formation during distraction osteogenesis were investigated. We have shown that the administration of alpha-tocopherol provided better results as far as the radiologic, scintigraphic and histopathologic evaluations were concerned.

Oxygen-derived free radicals are highly toxic molecules that produce cellular damage by causing both structural and functional impairment in almost all components of the cell, but mainly the cell membrane. They initiate a chain reaction leading to cell membrane damage via lipid peroxidation, thereby causing cell lysis [25]. Alpha-tocopherol is a natural macromolecule that acts as a biological antioxidant in the cell membranes, inhibiting lipid peroxidation by scavenging peroxy and alkoxy radicals and thus breaking chain reactions [26, 27].

Distraction osteogenesis is widely used for the treatment of various challenging musculoskeletal disorders. Prolonged time spent with external fixation is one of the disadvantages that can cause complications such as pin tract infection, loosening, muscle weakness and contractures [28]. Furthermore, prolonged framing time decreases the compliance of patients and causes psychological and behavioral problems [29].

Distraction osteogenesis is considered intramembranous ossification, which can be assumed to be a special form of fracture healing [12]. During the initial ischemic stage, considerable amounts of oxygen-derived free radicals are produced due to the activation of inflammatory cells [30, 31]. Likewise, Prasad et al. measured the predictors of oxidative stress in fracture patients and found that oxidative stress was directly proportional to the number of fractures, and that it peaked at the 3th week after the fracture and continued until the 4th week [32].

On the other hand, various experimental studies have been carried out to accelerate and shorten fracture healing with the administration of antioxidants. Göktürk et al. demonstrated that the administration of zymosan—which induces oxygen-free radicals through the stimulation of NADPH oxidase in polymorphonuclear leukocytes—impaired fracture healing in a rat model [17]. Yilmaz et al. have demonstrated the positive effects of ascorbic acid, a well-known antioxidant, on fracture healing [21]. Moreover, there are also studies that have shown the beneficial effects of alpha-tocopherol on fracture healing, whereas its effects on distraction osteogenesis have not been investigated [18–20]. Therefore, to our best knowledge, our study is the first report in this regard.

The fact that serum TAC values were not significantly different from those of the control group on the 5th day of our study, but they were significantly greater on the 30th day, suggests that alpha-tocopherol exerts a favorable effect during the ischemic stage but not during the inflammatory period. Radiologic and histologic evidence of callus formation and maturation were also found to be directly proportional to serum TAC values in the experimental group. It is also worth noting that decreasing the ischemic stage using antioxidants would not only induce osteoblastic activity but it would also impede the osteoclastic resorption of newly formed bone due to oxygen-derived free radicals.

Overall, based on our study, we may conclude that the administration of supplemental alpha-tocopherol in patients treated with distraction osteogenesis may shorthen the framing time and increase the quality of the regenerated bone. Further clinical studies are necessary to check its effects on humans and also to ascertain whether it should be used prophylactically or continuously until the end of the consolidation period. However, when compared with normal fracture healing, alpha-tocopherol may be much more effective at decreasing the repetitive ischemic cycles that are produced during distraction osteogenesis.
